# Acute Kidney Injury Associated with Cardiac Surgery: a Comprehensive Literature Review

**DOI:** 10.21470/1678-9741-2019-0122

**Published:** 2020

**Authors:** Amer Harky, Mihika Joshi, Shubhi Gupta, Wan Yi Teoh, Francesca Gatta, Mostafa Snosi

**Affiliations:** 1 Department of Cardiothoracic Surgery, Liverpool Heart and Chest, Liverpool, UK.; 2 Department of Cardiology, Countess of Chester Hospital, Chester, UK.; 3 School of Medicine, University of Liverpool, Liverpool, UK.

**Keywords:** Acute Kidney Injury, Cardiac Surgical Procedures, Postoperative Period, Postoperative Complications

## Abstract

**Objective:**

To comprehensively understand cardiac surgeryassociated acute kidney injury (CSA-AKI) and methods of prevention of such complication in cardiac surgery patients.

**Methods:**

A comprehensive literature search was performed using the electronic database to identify articles describing acute kidney injury (AKI) in patients that undergone cardiac surgery. There was neither time limit nor language limit on the search. The results were narratively summarized.

**Results:**

All the relevant articles have been extracted; results have been summarized in each related section. CSA-AKI is a serious postoperative complication and it can contribute to a significant increase in perioperative morbidity and mortality rates. Optimization of factors that can reduce CSA-AKI, therefore, contributes to a better postoperative outcome.

**Conclusion:**

Several factors can significantly increase the rate of AKI; identification and minimization of such factors can lead to lower rates of CSA-AKI and lower perioperative morbidity and mortality rates.

**Table:** 

Abbreviations, acronyms & symbols				
ACE	= Angiotensin-converting enzyme		IL-18	= Interleukin-18
ACEIs	= Angiotensin-converting enzyme inhibitors		KDIGO	= Kidney Disease Improving Global Outcomes
ACx	= Aortic cross-clamping		KIM-1	= Kidney injury molecule-1
AKI	= Acute kidney injury		L-FABP	= Liver fatty-acid binding protein
AKIN	= Acute Kidney Injury Network		MCPB	= Miniaturized cardiopulmonary bypass
ANP	= Atrial natriuretic peptide		NAC	= N-acetylcysteine
ARBs	= Angiotensin receptor blockers		NGAL	= Neutrophil gelatinase-associated lipocalin
BNP	= Brain natriuretic peptide		NMA	= Network meta-analysis
CABG	= Coronary artery bypass grafting		NSAIDs	= Nonsteroidal anti-inflammatory drugs
CCABs	= Cell-cycle arrest biomarkers		OPCAB	= Off-pump coronary artery bypass
CI	= Confidence interval		OR	= Odds ratio
CI-AKI	= Contrast-induced acute kidney injury		PRBC	= Packed red blood cell
CIN	= Contrast-induced nephropathy		RCT	= Randomised controlled trial
CKD	= Chronic kidney disease		RIFLE	= Risk, Injury, Failure, Loss of kidney function, and
CPB	= Cardiopulmonary bypass			End-stage kidney disease
CSA-AKI	= Cardiac surgery-associated acute kidney injury		RIPC	= Remote ischaemic preconditioning
CyC	= Serum cystatin C		ROS	= Reactive oxygen species
EPO	= Erythropoietin		RR	= Risk ratio
FDA	= Food and Drug Administration		RRT	= Renal replacement therapy
GDT	= Goal-directed therapy		SCr	= Serum creatinine
GFR	= Glomerular filtration rate		SIRS	= Systemic inflammatory response syndrome
HES	= Hydroxyethyl starch		suPAR	= Soluble urokinase plasminogen activator receptor
ICU	= Intensive care unit		TEA	= Thoracic epidural analgesia
IGFBP7	= Insulin-like growth factor binding protein 7		TIMP2	= Tissue inhibitor of metalloproteinases-2

## INTRODUCTION

Acute kidney injury (AKI) is a condition that is characterized by a sudden deterioration of kidney function as indicated by a reduced glomerular filtration rate (GFR)^[1]^. Cardiac surgeryassociated acute kidney injury (CSA-AKI) originates from cardiac surgery and percutaneous coronary interventions with prolonged intensive care stays leading to either renal injury and/ or high mortality rates postoperatively; cardiac surgery is also the second most common cause of AKI in the intensive care unit (ICU)^[1]^. Nephrotoxins, metabolic abnormalities, ischemia and reperfusion injury, pre-existing chronic diseases, inflammation, and oxidative stress all contribute to the development of AKI postoperatively^[2]^.

Depending on different classifications, the incidence of AKI post cardiac surgery varies from 5 to 43%, with 1 to 7% of them requiring dialysis^[3]^. The large variation in the incidence rate is dependent on the type of the surgical procedure performed, ranging from as high as 94% in heart transplantation to as low as 3% in thoracic surgery^[3]^. In addition, up to 52% of children are being diagnosed with AKI post cardiac surgery^[4]^, which can create huge socioeconomic burdens on clinical institutions such as the National Health Service, from the United Kingdom. Severe CSA-AKI is independently associated with 3-8-fold higher mortality, increased expenses, and lengthy ICU stays.

Due to the lack of a uniform definition of AKI, research into its detection and management strategies has been having difficulties in comparing the outcomes. The most frequently used classifications by researchers are Risk, Injury, Failure, Loss of kidney function, and End-stage kidney disease (RIFLE), Acute Kidney Injury Network (AKIN), and Kidney Disease Improving Global Outcomes (KDIGO). RIFLE and AKIN use criteria such as change in serum creatinine (SCr) level, increase of at least 1.5 times from the baseline, and urine output of < 0.5ml/kg/h for at least six hours^[5]^. KDIGO is based on the combination of RIFLE and AKIN criteria and has become a novel consensus classification for diagnosis of CSA-AKI; it has also been shown to have greater sensitivity in detection of AKI postoperatively than other classifications^[6]^.

Currently, AKI is diagnosed based mainly on sharp rises in SCr levels, however this can take up to 48 hours to raise and become clinically significant and, therefore, it is not an effective method of detecting AKI; hence, novel biomarkers are needed to allow for successful therapeutic interventions.

This review focuses on the pathophysiology, risk factors, diagnosis, management, and prevention of CSA-AKI.

## PATHOPHYSIOLOGY AND RISK FACTORS

**Fig. 1:**
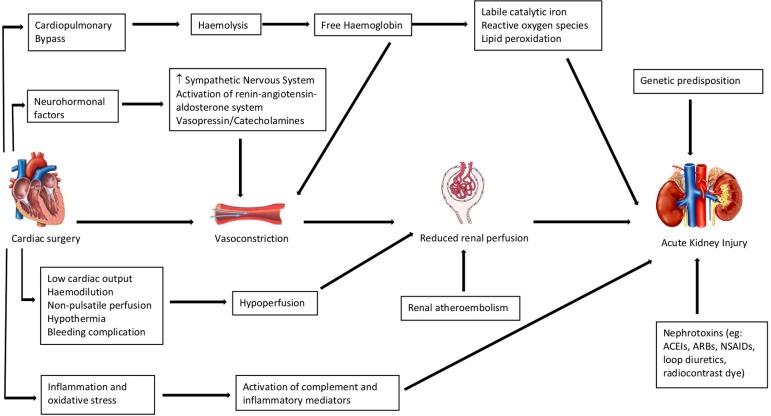
Pathophysiology of cardiac surgery-associated acute kidney injury. ACEIs=angiotensin-converting enzyme inhibitors; ARBs=angiotensin receptor blockers; NSAIDs=nonsteroidal anti-inflammatory drugs

The precise pathophysiology of CSA-AKI remains not completely understood. However, animal and human studies suggested that the mechanisms appear to be multifactorial, with each of these factors interacting with one another preoperatively, intraoperatively, and postoperatively. These include genetic predisposition, nephrotoxins, cardiopulmonary bypass (CPB)induced haemolysis, ischemic-reperfusion injury, complexity of cardiac surgery, oxidative stress, and inflammation (Figure 1)^[7]^.

Furthermore, CSA-AKI is a serious postoperative complication, which can lengthen recovery in up to 30% of patients and is the strongest predictor of mortality following surgery^[7]^. A complex interplay between patient and procedure-related factors can be attributed to the development of AKI postoperatively. Patients undergoing cardiac surgery are particularly susceptible due to the unique physiology and underlying procedures involved, including aortic cross-clamping (ACx) and CPB and the use of frequent transfusions and vasopressor^[7]^. Figure 1 highlights the pathophysiological mechanisms leading to development of AKI.

### Preoperative Risk Factors

Patients’ demographics including advancing age, race, and gender are all non-modifiable preoperative risk factors for developing AKI following cardiac surgery. Firstly, advancing age is associated with declining renal function and an associated reduction in estimated GFR, which compromises normal renal physiology and increases predisposition to developing AKI postoperatively. Secondly, studies have found an increased tendency for females to develop AKI compared with males^[8]^. Lastly, another study has reported better morbidity and mortality outcomes following development of postoperative AKI in Afro-Caribbean races, compared with their Caucasian counterparts^[9]^.

For patients undergoing cardiac surgery, baseline comorbidities and functional status are two extremely important factors, which can determine the likelihood of developing AKI postoperatively. Patients with comorbidities, such as chronic obstructive pulmonary disease, diabetes mellitus, congestive cardiac failure, and preexisting chronic kidney disease (CKD), are all likely to be predisposed to developing renal insult following cardiac procedures. This could be due to impaired renal perfusion, endothelial insult, and decreased renal reserve. Furthermore, patients with such comorbidities are frequently administered a variety of nephrotoxic medications, such as nonsteroidal anti-inflammatory drugs (NSAIDs), angiotensin-converting enzyme inhibitors (ACEIs), and angiotensin receptor blockers (ARBs), all of which further adversely alter glomerular perfusion and may induce nephropathy. Hence, this leads to increased likelihood of impaired renal function and higher susceptibility towards developing AKI postoperatively.

Preoperative cardiac insult is another factor that has been associated with the development of postoperative AKI. Mao et al.^[1]^ describes the risk associated with low cardiac output in either the pre, intra, or postoperative period and the increased likelihood of developing postoperative AKI due to reduced perfusion pressures and global renal ischaemic insult. This can be attributed to sympathetic hyperactivity and the simultaneous activation of the renin-angiotensin-aldosterone system, which leads to renal vasoconstriction and, hence, reduced renal perfusion^[1]^. Low cardiac output and insult may also warrant the use of preoperative intra-aortic balloon pump, which has also been associated with development of postoperative AKI, as suggested by Rosner et al.^[8]^

Recently, genetic predisposition to AKI has been studied. Stafford Smith et al. conducted an unbiased genome-wide associated study which identified two new loci, namely *rs13317787* in *GRM7|LMCD1-AS1* intergenic region (3p21.6) and r*s10262995* in *BBS9* (7p14.3), that increased the risk of AKI following coronary artery bypass grafting (CABG)^[10]^. The authors also speculated that their findings may become a predictive tool to improve individualised AKI risk stratification in cardiac surgery patients. According to a genetic polymorphisms study, the apolipoprotein E, a cardinal protein for lipoprotein metabolism, tissue repair, and immunomodulation, was associated with AKI, and the only genotype that possessed AKI protective effect was the ε4 allele^[11]^.

Patients undergoing cardiac surgery often have had reduced renal blood flow due to recent myocardial infarction or valvular disease with reduced cardiac output. Administration of nephrotoxic medications, such as loop diuretics, NSAIDs, ACEIs, ARBs, and antibiotics (*e.g.* aminoglycoside or beta-lactam inhibitors), prior to or after surgery could further enhance the likelihood of developing AKI. The use of antibiotics can lead to acute interstitial nephritis or direct injury whereas ACEIs and ARBs can cause volume depletion and inhibition of renal efferent arteriolar vasoconstriction, respectively^[7]^. Multiple studies have demonstrated a directly causal relationship between the use of intravenous radiocontrast for diagnosis (angiography or ventriculography) and the development of contrast-induced nephropathy (CIN). This is affected by several factors, including type and dose of contrast medium, and patients’ demographics, such as age, gender, hydration status, and underlying comorbidities, including CKD^[12]^.

Another potential nephrotoxin is free haemoglobin resulting from CPB-induced haemolysis. CPB haemolyzes erythrocytes and leads to the generation of intravascular free haemoglobin, which depletes circulating haptoglobin and directly injures renal endothelium and tubular epithelium through iron-facilitated free radical oxidation^[1]^.

Another common cause of CSA-AKI is renal atheroembolism. Preoperative procedures, such as cardiac catheterization, aortic and left atrial manipulation, aorta cannulation, and ACx, could lead to deposition of emboli in renal artery, further exacerbating ischaemia and triggering inflammatory response. Other factors that could play a role in the reduction of renal blood flow leading to diminished glomerular filtration rate include increased sensitivity to sympathetic nervous system, activation of renin-angiotensin-aldosterone cascade, and circulating vasopressin or catecholamines^[7]^. Table 1 highlights the frequently associated preoperative risk factors with the development of AKI post cardiac surgery.

**Table 1:** Preoperative risk factors associated with development of acute kidney injury.

Race
Gender: female > male
Advancing age
Genetics
Comorbidities:
Peripheral vascular disease
Chronic obstructive pulmonary disease
Congestive cardiac failure
Pre-existing renal disease
Diabetes
Anemia
Chronic liver disease
Previous cerebrovascular accidents
Generalized atherosclerotic disease
Preoperative use of intra-aortic balloon pump

### Intraoperative Risk Factors

O’Neal et al.^[7]^ suggest that ACx, CPB, and the frequent use of blood transfusions and vasopressors are unique to cardiac surgery. However, such factors have repeatedly been reported to increase the risk of developing AKI following such operations^[13]^.

#### A. Ischemic Injury or Inadequate Renal Perfusion and Reperfusion

Even though the kidneys receive approximately a quarter of the cardiac output, the renal blood flow to the medulla is low compared to the cortex where the glomerular filtration and reabsorption of solute occurs normally. The shunting of blood from arterial to venous vasa recta results in the deficiency of oxygen in the high metabolic demands region, the outer medulla, which corresponds to the thick ascending limb of the loop of Henle. This region is responsible for the generation of osmotic gradient by active reabsorption of sodium, which requires high oxygen consumption. In addition, the medullary partial pressure of oxygen is lower than that of the cortex, 10 to 20 mm Hg and around 50 mm Hg, respectively. Hence, renal medulla and corticomedullary junction are more vulnerable to hypoxic and ischemic damages when there is any factor in cardiac surgery that affects the renal perfusion.

#### B. Cardiopulmonary Bypass

CPB exposes red blood cells to artificial surfaces within the CPB circuit causing their haemolysis^[8]^. The breakdown of these cells results in haemoglobin deposition within the intratubular vasculature of the kidneys. This combined with the oxidative damage caused by iron has been thought to contribute to AKI development. Obialo et al.^[14]^ have also suggested that ACx and aortic de-clamping during CPB can also result in subsequent ischaemia and contribute to reperfusion injury along with systemic embolization. The use of CPB circuit is associated with haemodilution due to CPB machines being primed with a minimum of 1.5-2 L non-hematic crystalloid/colloid fluids. This results in a reduction of hematocrit concentrations of about < 20%, which causes a reduction in the oxygen-carrying capacity of blood and, hence, ischaemia to end organs.

Lastly, the role of pulsatile *versus* non-pulsatile flow has been discussed as a contributory factor to AKI postoperatively. Mao et al.^[1]^ have also demonstrated that off-pump cardiac surgery offers more physiological renal perfusion, decreases the systemic inflammatory response, and causes less systemic embolization, which are common complications associated with on-pump cardiac surgery. Pulsatile flow was developed to increase the mechanical energy transmitted via CPB circuit, which results in increased release of vasodilatory substances, less systemic vascular resistance, and improved end-organ perfusion^[1]^. However, this remains a controversial risk factor as there is no definitive evidence base that suggests that off-pump surgery is more beneficial compared with on-pump surgery.

#### C. Inflammation

The conventional CPB on-pump CABG provokes systemic inflammation response and has been associated with elevated postoperative plasma concentration of pro-inflammatory cytokines compared with off-pump coronary artery bypass (OPCAB), leading to increased mortality. A systematic review by Cheungpasitporn et al.^[15]^, which included 33 randomised controlled trials (RCTs), demonstrated a protective effect of OPCAB in lowering AKI risk, but the finding was contraindicated by the largest multicenter RCT (the CORONARY trial)^[16]^, which found no significant difference between the OPCAB and on-pump CABG groups. In conclusion, the use of OPCAB in reducing incidence of AKI still remains unclear in the current reported literature.

The mechanisms of increased inflammatory response are not well defined, but the complement activation during CPB is believed to occur mainly through the alternative pathway when the blood is in contact with the surface of extracorporeal circuits^[17]^. In a prospective study, Bruins et al. reported a biphasic complement activation in cardiac surgery patients, which activated not only during CPB but also during the first five days thereafter, that increased C-reactive protein levels, contributing to the second activation^[17]^.

Other stimuli, such as endotoxemia, tissue injury, operative trauma, and pre-existing left ventricular dysfunction, could possibly play a role in activation of the immune system. Proinflammatory cytokines and free radicals are produced, leading to renal tubular injury, which is further enhanced by the activation of neutrophils, macrophages, and migration of lymphocyte to renal parenchyma^[18]^. Ischemia-reperfusion injury occurs and induces the production of reactive oxygen species (ROS). The ROS, in turn, activate the up-regulation of pro-inflammatory transcription factors, including nuclear factor kappa B, inflammatory mediators, and adhesion molecules, leading to cellular injury and AKI^[8]^. Table 2 demonstrates intraoperative risk factors that can influence the development of postoperative AKI.

**Table 2:** Intraoperative risk factors associated with development of acute kidney injury.

Increased cardiopulmonary bypass time
Type of procedure:
Valvular surgery *vs*. CABG *vs*. valvular surgery and CABG
Off-pump *vs*. on-pump
Aortic cross-clamping duration
Nephrotoxic drug use:
Angiotensin-converting enzyme inhibitors
Nonsteroidal anti-inflammatory drugs
Angiotensin receptor blockers
Exposure to contrast agents/dyes
Emergency surgery
Renal hypoperfusion
Hypothermia

CABG=coronary artery bypass grafting

### Postoperative Risk Factors

Postoperatively, predominant factors influencing the development of AKI include haemodynamic instability, nephrotoxic, inotropic and vasoconstrictor drugs, and systemic inflammation^[8]^. The presence of congestive heart failure or left ventricular dysfunction postoperatively are significant factors contributing to development of AKI due to the impaired ejection fraction and perfusion pressure to the kidneys^[7]^. Moreover, preexisting anaemia or the development of anaemia following surgery can also results in AKI due to the reduced oxygencarrying capacity of red blood cells and ischaemic insult to the renal system. Table 3 is a summary of key postoperative factors contributing to the development of AKI post cardiac surgery.

**Table 3:** Key postoperative factors contributing to the development of acute kidney injury.

Nephrotoxic drug use	
Haemodynamic instability	
Reduced cardiac output	
Blood transfusion	
Sepsis/septic shock	
Inotropic drugs	
Vasoconstrictor drugs	
Anaemia	

Khan et al. studied the role of packed red blood cell (PRBC) blood transfusion in causing CSA-AKI in 1210 adult patients undergoing cardiac surgery. The study demonstrated that blood transfusion of at least two units is associated with AKI, which caused doubling of SCr from the preoperative value and doubling of postoperative urinary novel biomarkers, such as interleukin-18 (IL-18) and neutrophil gelatinase-associated lipocalin (NGAL). However, the authors acknowledged that the study was limited by its observational nature, no set transfusion trigger, and variable age of PRBC; also, a direct potential causal association between PRBC and AKI could not be confirmed^[19]^.

## BIOMARKERS IN THE SETTING OF AKI

In the clinical practice, SCr remains the diagnostic standard of AKI. According to KDIGO classification, if SCr increases by ≥ 0.3 mg/dl (26.5 µmol/l) within 48 hours, SCr is 50% higher than the baseline within first seven days, or urine output is below 0.5 ml/kg/hour for six hours, the patient is considered to have AKI^[20]^. However, the classic markers (SCr, urea, and urine output) are insensitive and nonspecific to renal injury. SCr often leads to delaying of diagnosing AKI as it can only be detected 48 to 72 hours after cardiac surgery, when glomerular filtration is already substantially reduced. SCr is not considered a robust marker for timely diagnosis in AKI and does not provide enough time for therapeutic treatment to work as it is subjected to several nonrenal factors, such as gender, age, fluid therapies, drugs, muscle metabolism, medical conditions, and other circumstances^[19]^.

Since SCr has various limitations, scientists have discovered the theoretical advantage of a panel of novel serum and urinary biomarkers that may closely reflect the early detection, even without the concurrent renal dysfunction, and prognostication of CSA-AKI. NGAL, the most extensively studied substance, is reported as an early biomarker of AKI after CPB, which increases 25-fold within two hours and declines six hours after CPB^[21]^. Urinary IL-18, a pro-inflammatory cytokine, raised at four to six hours after cardiac surgery, peaked at over 25-fold at 12 hours and remained significantly elevated up to 48 hours after surgery^[21]^. NGAL and IL-18 could be early predictors of important outcomes such as length of hospitalisation, need for dialysis, and mortality^[21]^.

In 2014, the United States Food and Drug Administration (FDA) has approved the production of tissue inhibitor of metalloproteinases-2 (TIMP2) and insulin-like growth factor binding protein 7 (IGFBP7) markers, involved in growth phase cell-cycle arrest of tubular epithelial cells, for their usefulness in the early detection of moderate to severe AKI defined as KDIGO stages 2 and 3. The product of both markers can be detected as early as four hours after surgery, and a decline in these markers was the strongest predictor of renal recovery^[22]^. TIMP2 and IGFBP7 can be a bedside test as they are easily measured by Point of Care kit (NephroCheck) approved by FDA^[22]^.

Serum cystatin C (CyC) is a low molecular weight protease inhibitor that is rapidly filtered by glomerulus. CyC reflects changes in GFR as the level increases when GFR decreases. CyC has also been found to have a better predictive value of AKI when compared with the conventional markers as it detects AKI two days earlier than creatinine^[23]^.

Other promising urine proteins, such as kidney injury molecule-1 (KIM-1) and liver fatty-acid binding protein (L-FABP), have been commonly introduced and investigated in animal models and preliminary human studies^[21]^. In adults, KIM-1 peaked two days after surgery, whereas L-FABP peaked within six hours after surgery^[21]^. KIM-1 could be helpful in differentiating ischemic AKI from prerenal azotemia and CKD.

However, the superiority of one biomarker over another and the cost effectiveness of using new biomarkers remain unclear. Given the multifactorial pathophysiology of CSA-AKI, clinicians could use the clinical assessment of patients with combination of these novel AKI biomarkers as a part of a panel for early prediction of AKI. Table 4 is a summary of the key biomarkers.

**Table 4:** Novel biomarkers for prediction of CSA-AKI.

Biomarker	Description	Advantages	Disadvantages
Soluble urokinase plasminogen activator receptor (suPAR)	Serum biomarker detected in body fluids like blood, urine, peritoneal fluids, and cerebrospinal fluids	- Preoperative detection of AKI - suPAR is a more sensitive detector than serum creatinine as suPAR levels are elevated in AKI patients when creatinine levels are not	- A reliable cutoff value for suPAR levels was not determined
Neutrophil gelatinase-associated lipocalin (NGAL)	NGAL is an iron-transporting glycoprotein which gathers in the kidney tubules and urine after nephrotoxic and ischemic insult	- Levels increase rapidly after renal ischemia-reperfusion injury - Renoprotective role in regeneration of kidney tubule	- Influenced by non-renal factors such as age, anaemia, cancer, CKD, and inflammatory conditions - Urine sample difficult to obtain in patients with severe oliguria
Interleukin-18	24kDa precursor, produced by various tissues such as monocytes, macrophages, and proximal tubular epithelial cells	- Biomarker with risk stratification potential, assist with stratification of patients based on severity of AKI - Mediator of ischemic reperfusion injury	- Urine sample difficult to obtain in patients with severe oliguria - Induces inflammation of kidney tubule
Galectin-3	Beta-galactoside-binding lectin, which is a regulator of inflammation and tissue fibrosis	- Preoperative detection of AKI	- Associated with renal fibrosis
Tissue inhibitor of metalloproteinases-2 (TIMP2) and insulin-like growth factor binding protein 7 (IGFBP7)	Soluble protein expressed by kidney involved in cell-cycle arrest during earliest phases of cellular stress and tubular injury	- Risk stratification of patients into high and low risk can be done, it is a better risk stratification than standard clinical assessment	- Oliguria impedes urine sampling of TIMP2 and IGFBP7 - There is an association between elevated CCABs and diabetes which might reflect subclinical, subacute, or chronic diabetesassociated renal injury and create a major cofounder to the predictive value for CSA-AKI
Kidney injury molecule-1 (KIM-1)	Immunoglobulin superfamily transmembrane receptor expressed in tubular kidney injury to help in removal of necrotic and apoptotic debris	- Urinary KIM-1 increased rapidly in models of ischemic AKI	- Influenced by non-renal factors such as sepsis, contrast media, age, diabetes mellitus, hypertension, and atherosclerosis - Role in carcinogenesis of renal cell carcinoma
Netrin-1	Small protein which plays an important role in formation of new blood vessels, cell adhesion, and tumorigenesis	- Protective role in promotion of kidney epithelial cell proliferation and inhibition of apoptosis	- High in patients with ischemia, contrast agents, drugs, and sepsis, hence, association with AKI might not be independent
Growth-differentiation factor-15	Expressed in many organs such as heart, lung, liver, and intestines, and is an independent predictor of postoperative renal dysfunction	- Preoperative detection - Superior predictive ability compared to risk scores such as EuroSCORE	- Marker is also associated with mortality and morbidity, hence, association with AKI might not independent

AKI=acute kidney injury; CCABs=cell-cycle arrest biomarkers; CKD=chronic kidney disease; CSA-AKI=cardiac surgery-associated acute kidney injury

## PREVENTION OF AKI

### Preoperative Prevention Strategies

#### A. Renal Recovery to Prevent Contrast-Induced AKI

The use of contrast agents frequently in clinical procedures prior to cardiac surgery is known to be nephrotoxic and also to promote development of contrast-induced AKI (CI-AKI), depending on factors such as type and dose of contrast agent, underlying CKD, age, and hydration status. Intravenous isotonic crystalloid solutions could decrease CI-AKI risk; however, excessive hydration using this medium may increase the incidence of CI-AKI. Cardiac surgery should be delayed whenever possible to prevent increased risk of AKI and to allow renal recovery as suggested by Medalion et al.^[24]^

#### B. Avoidance of Intraoperative Anaemia and Haemolysis

Low preoperative and intraoperative haemoglobin levels are associated with AKI post cardiac surgery. Karkouti et al.^[25]^ reported that the risk of AKI is independent of the lowest haemoglobin values but it is significantly increased when haemoglobin decreases more than 50% below baseline. An association between low perioperative haemoglobin concentrations and postoperative AKI in non-cardiac surgeries exists^[26]^, and may well apply for cardiac surgeries as well. This indicates that preoperative haemoglobin levels should be optimised preoperatively.

#### C. Choice of Surgery Technique

In a systematic review of 33 RCTs with 17322 patients, the authors reported a pooled risk ratio (RR) of AKI in the off-pump *vs*. on-pump CABG groups to be 0.87 (95% confidence interval [CI], 0.77-0.98)^[27]^. However, off-pump CABG may not have any effect on death, stroke, and myocardial infarction as concluded by Lamy et al.^[16]^. On the other hand, Reents et al.^[27]^ have found no significant difference in the incidence of AKI. Conflicting results provide unclear evidence to the superiority of off-pump CABG in reduction of AKI rate.

### Intraoperative Prevention Strategies

#### A. Glucose Homeostasis

Hyperglycaemia is associated with increased incidence of AKI, mortality, and morbidity^[28]^. Van den Berghe et al.^[28]^ first investigated the effects of glucose homeostasis and found out that intensive insulin therapy (blood glucose level of 80-110 mg/dL) relative to conventional control (180-200 mg/dL) was associated with decreased morbidity and mortality. However, subsequent studies have failed to produce similar results^[29]^. Song et al.^[29]^ did demonstrate an association between intraoperative glucose concentration > 150 mg/dl and AKI risk after OPCAB; however, they have failed to reaffirm the significance of intensive insulin therapy (< 110 mg/dL) on AKI. KDIGO recommends that blood glucose levels should not exceed 150 mg/dL, but that insulin therapy should not be used to lower blood glucose to less than 110 mg/dL.

#### B. Goal-Directed Therapy (GDT)

GDT is associated with a reduction in the incidence of AKI, renal replacement therapy (RRT), ICU stay, and hospital stay, as well as decreased risk of renal dysfunction and reduced mortality^[30]^. Magruder et al.^[31]^ investigated the effects of goaldirected perfusion therapy on AKI in a pilot study where AKI incidence was 23.9% in control patients *vs*. 9.1% in the GDT group, indicating an association between reduced AKI and GDT.

#### C. Epidural Anaesthesia

Thoracic epidural analgesia (TEA) leads to a significantly decreased risk of AKI development post CABG surgery^[32]^. However, Svircevic et al.^[33]^ produced conflicting results suggesting the risk of TEA usage in a patient who may require full heparinisation. Nonetheless, the use of epidural analgesia in cardiac surgery has been associated with a reduction in mortality^[34]^.

#### D. Remote Ischaemic Preconditioning

Remote ischaemic preconditioning (RIPC) has been hypothesised to prevent organ dysfunction in cardiac surgery patients; however, the use of RIPC as a preventative measure is still controversial. RIPC has failed to show significant reduction of the incidence of AKI after cardiac surgery^[35]^.However, Zarbock et al.^[36]^ have found out that RIPC significantly reduced the 3-month incidence of mortality, need for RRT, and renal injury in high-risk patients undergoing cardiac surgery. Available evidence makes it unclear whether RIPC is effective against AKI.

#### E. Fluid Management

The type of fluid administered may affect the rate of development of AKI. Martin et al.^[37]^ found crystalloids to be less efficient at stabilising resuscitation endpoints than colloids. Within crystalloid solutions, isotonic saline administration is associated with increased AKI risk due to excess chloride. Buffered crystalloid fluid therapy produces no difference in the risk of AKI when compared to saline solution^[38]^. However, a systematic review of 6253 patients proposed that postoperative fluid resuscitation with balanced crystalloids reduced incidence of AKI when compared to physiologic saline solutions^[39]^. Hydroxyethyl starch has been associated with an increased risk of AKI; hence, it should be considered carefully in high-risk patients^[40,41]^. Table 5 is a summary of the key studies comparing both solution types.

**Table 5:** Crystalloid and colloid solutions in prevention of AKI.

Study	Objective	Definition	Intervention	Results	Conclusion
Myburgh et al^[40]^.	To investigate the efficacy of hydroxyethyl starch (HES) for fluid resuscitation and effects on renal function.	RIFLE	6% HES in 0.9% saline *vs*. 0.9% saline solutions until discharge, death, or 90-day randomisation.	AKI incidence: 34.6% in HES group vs. 38% in saline group. Renal replacement therapy (RRT) incidence: 7.0% in HES group *vs*. 5.8% in saline group.	HES provided no clinical benefit and resulted in increased rate of RRT.
Haase et al.^[68]^	To evaluate the efficacy of prophylactic bicarbonate-based infusion to reduce the incidence of AKI in patients undergoing open heart surgery.	RIFLE	Sodium bicarbonate (5.1 mmol/mL) *vs*. saline solution started before surgery until 24 hours after the end of procedure.	AKI incidence: 47.7% in sodium bicarbonate group *vs*. 36.4% in control group.Mortality in bicarbonate group was 6.3% *vs*. 1.7% in control group	Greater mortality and no prophylactic effect associated with sodium bicarbonate solution.
Soh et al.^[41]^	To investigate the preoperative administration of sodium bicarbonate in postoperative AKI prevention after off-pump coronary revascularization.	AKIN	Sodium bicarbonate (0.5 mmol kg-1 for 1 h upon induction of anaesthesia followed by 0.15 mmol kg-1 h-1 for 23 h) *vs*. 0.9% saline.	Incidence of AKI: 21% in bicarbonate group *vs*. 26% in control group. More patients required prolonged mechanical ventilation (> 24 h) relative to the control group.	Perioperative use of sodium bicarbonate did not reduce incidence of AKI and might be associated with a needed for prolonged ventilatory care.
Young et al^[38]^.	To investigate the effect of buffered crystalloid compared with saline on renal complications in patients in the ICU.	RIFLE	Double-blind cluster randomised double-crossover trial in 4 ICUs. Participants were assigned Plasma-Lyte 148 or saline solution for alternating treatment blocks of 7 weeks over 28 weeks.	9.6% developed AKI within 90 days in buffered crystalloid group *vs*. to 9.2% in saline group.	Use of crystalloid fluid therapy was ineffective in prevention of AKI in the ICU.

AKI=acute kidney injury; AKIN=Acute Kidney Injury Network; ICU=intensive care unit; RIFLE=Risk, Injury, Failure, Loss of kidney function, and End-stage kidney disease

Perioperative fluid overload is also associated with increased severity of AKI and increased mortality post cardiac surgery^[42]^. In a recent study by Bhaskaran et al.^[43]^, they found out that perioperative use of chloride-restricted intravenous fluids is associated with statistically significant lower incidence of AKI stage I, whilst chloride-liberal intravenous fluids are associated with hypochloremia acidosis and increased incidence of AKI stage I post CABG. Hence, continued chloride-restricted fluids could possibly reduce incidence of AKI and result in better clinical outcomes.

Positive fluid balance management is strongly associated with a higher AKI rate; however, there is no association between volume of negative fluid balance and AKI incidence^[44]^.

#### F. Miniaturized CPB

Initially, the miniaturized cardiopulmonary bypass (MCPB) systems have been developed to render on-pump cardiac surgeries more efficient and the preliminary reported incidence of AKI was lower in patients that had MCPB than those with conventional CPB (29% *vs*. 41%)^[45]^. However, in the study by Chew et al.^[46]^, they have identified similar rates of AKI in both cohorts and thus the superiority of either technique remains debatable^[46]^.

#### G. Duration of CPB

Several studies have found out that there is a potential direct association between prolonged CPB and ACx times and development of AKI. Karim et al.^[47]^ reported a positive association between increasing ACx and CPB times and incidence of CSA-AKI; they found out that CPB time > 70 min increased the risk of CSA-AKI by an odds ratio (OR) of 4.76 when compared to CPB time > 140 min with an OR of 6.30. In a meta-analysis of nine studies by Kumar et al.^[47]^, they concluded that longer CPB and ACx times are strongly associated with higher incidence of AKI; however, they were unable to determine the safe cutoff time. Nevertheless, the consensus is that the shorter the CPB or ACx time, the less likely development of CSA-AKI.

#### H. Haemoadsorption Device

This technique uses a cytokine adsorber to eliminate inflammatory mediators, *i.e*., cytokines to restore immune homeostasis. Since inflammation is associated with the development of CSA-AKI, haemoadsorption, mediating this response, can potentially reduce the risk of AKI. Studies have reported that the haemoadsorption device used in cardiac surgeries involving CPB leads to cytokine reduction, improves haemodynamic stability and organ function^[48]^, and reduces postoperative systemic inflammatory response syndrome (SIRS).

## MANAGEMENT OF AKI

### A. Conservative Management

Following development of AKI post cardiac surgery, management options include conservative treatment focused on using pharmacological interventions or the use of RRT.

Prevention of AKI development following cardiac surgery remains the optimal management; this includes optimization of multiple factors in the pre, intra, and postoperative periods. Rosner MH et al.^[8]^ recommended identification of highrisk patients preoperatively using comorbidities and the abovementioned scoring systems. Several measures have been demonstrated to be useful, such as withholding nephrotoxic medications like ACEI, ARBs, and NSAIDs to prevent hypotension during bypass, as well as metformin to prevent lactic acidosis.

Intraoperative measures should aim to improve renal reserve by improving perfusion pressures and, hence, reducing ischaemic insult to the kidneys, especially in those patients who are known to have pre-existing CKD. For patients with a background of CKD requiring dialysis, they can undergo dialysis 24 hours pre and postoperatively. Moreover, intraoperative hemofiltration plays a role in decreasing systemic fluid overload and improving respiratory function in patients with background congestive heart failure. KDIGO have also highlighted the importance of strict glycaemic control as well as avoidance of contrast agents in high-risk patients.

Loop diuretics can be used if oliguric renal failure persists despite optimisation of the abovementioned factors. This intervention can often convert oliguric to non-oliguric renal failure, preventing tubular damage, decreasing oxygen consumption, and potentially improving urine output^[8]^. Additionally, it is thought that patients who respond to such diuretic treatment often have better physiological reserve in their kidneys and will exhibit a reduction in their SCr quicker than those who don’t respond to loop diuretics.

Additionally, it is important to optimise haemodynamics in the pre, intra, and postoperative periods. This includes reducing preload, increasing afterload, and improving the contractility of the heart to increase the cardiovascular efficacy during surgery. There is ongoing debate as to the use of colloids *vs.* crystalloids in doing so; Meersche et al. recommend the use of crystalloid solutions with balanced electrolytes to mimic normal homeostatic physiology as closely as possible whilst tightly regulating blood pressure^[13]^. This prevents renal ischaemia and ensures adequate perfusion throughout, preventing renal tubular and parenchymal injuries. On the other hand, studies have also suggested that the use of colloids is the best strategy to maintain intravascular volume and haemodynamic stability. Paradoxically, studies also advise fluid restricting patients, as Stewart et al. have previously demonstrated a reduction in ICU stay and more ventilator-free days in cardiac surgery patients who underwent conservative fluid management^[49]^.

### B. Renal Replacement Therapy

For patients with severe AKI or oliguric renal failure, RRT is the only effective therapeutic intervention; this includes the use haemodialysis, haemofiltration, or haemodifiltration^[13]^. The indications for RRT have been extensively studied, yet no definitive evidence has been established regarding the most appropriate criteria or timing to initiate or discontinue RRT. Sun et al. have demonstrated a correlation between the initiation of early RRT and reduced mortality, improved survival rates, and an overall reduction in ICU length of stay^[50]^. Furthermore, early intervention also reduces risk of hyperkalaemia, uraemia, and acidaemia, whilst also providing haemodynamic stability.

#### 1. Indications for RRT

Some of the urgent indications to initiate RRT include azotemia, oliguria, and pulmonary oedema, with or without peripheral oedema that is unresponsive to diuretic therapy^[51]^. Moreover, Palevsky et al.^[52]^ also suggested the presence of severe hyperkalaemia, acid-base imbalances, encephalopathy, and uraemic pericarditis to be significant pathologies that should direct use RRT^[52]^.

#### 2. RRT Failure Risk Factors

Older age, intra and postoperative blood transfusions, nonemergent surgery, gender, and increased preoperative SCr and uric acid levels have all been attributed as risk factors for failure to respond to RRT^[53]^. RRT also exposes patients to a variety of risks, including the possibility of developing encephalopathy, aspiration, coagulopathies, such as bleeding disorders and plate dysfunction, and bacteraemia. However, these risks are outweighed by the benefits of achieving haemodynamic stability with use of RRT and potential improvement in renal function.

## PHARMACOLOGICAL PROTECTION

Several pharmacological approaches to prevent CSA-AKI have been studied and published, but, at present, there is no clear evidence to support the use of any pharmacological drug routinely in preventing AKI. Table 5 is a summary of the key studies about the pharmacological protection against development of AKI in cardiac surgery patients.

Dexmedetomidine, a highly selective central alpha2 adrenoreceptor agonist, was found to reduce the incidence of AKI in several studies. Recently, Liu et al. conducted a meta-analysis of 10 RCTs, involving 1575 patients undergoing cardiac surgery, to compare the effect of dexmedetomidine with placebo and other drugs on the AKI risk and mortality^[54]^. They reported an association of dexmedetomidine in decreasing postoperative AKI risk, but no significant decrease in other parameters such as duration of mechanical ventilation and length of hospital or ICU stay. Balkanay et al. performed a triple-blinded RCT and demonstrated marked effects of dexmedetomidine on renoprotection and decreasing NGAL concentration after CABG under CPB in a dose-dependent pattern^[55]^. However, to date, the optimal dose of dexmedetomidine to improve renal function after cardiac surgery remains uncertain. On top of the lipid-lowering properties, statins have anti-inflammatory, antioxidant, and antithrombotic properties, thus aiding in improving the endothelial function. All these properties proposed that statin is likely to be renoprotective. A retrospective study by Billings et al.^[55]^ found out that early postoperative statin administration may play a role in preventing AKI. On the contrary, Lewicki et al.^[56]^, who conducted a systematic review with 662 participants, led to a conclusion that empirical statin therapy did not improve renal function or mortality. Large high-quality RCTs are warranted to establish the safety and efficacy of statins to prevent AKI after cardiac surgery.

Renal vasodilators, such as fenoldopam, natriuretic peptide, and levosimendan, have been verified by the network metaanalysis (NMA) performed by Chen et al.^[57]^ for their effectiveness in increasing renal blood flow and reducing AKI risk after cardiac surgery. However, the current guideline from KDIGO expert committee does not include fenoldopam and natriuretic peptide^[20]^.

Fenoldopam, a dopamine-1 receptor partial agonist, may have renoprotective function due to its vasodilatory and natriuretic effect in potentially reversing renal hypoperfusion. Ranucci et al.^[58]^ performed a double-blind RCT including 80 patients undergoing complex cardiac surgery. The authors found out that patients treated with fenoldopam had higher oxygen delivery during CPB, lower AKI risk, and major morbidity rates than the placebo group^[57]^. In contrast, one recent metaanalysis in cardiac surgery and major surgery demonstrated the association of fenoldopam in reducing AKI significantly, but with no impact on the requirement of RRT, mortality, and length of ICU or hospital stays^[58]^.

Atrial natriuretic peptide (ANP) and brain natriuretic peptide (BNP) suppress the renin-angiotensin-aldosterone system and induces renal arterial vasodilation, which reduces renal ischemiareperfusion injury^[59]^. Sezai et al.^[60]^ reported that prophylactic use of ANP during cardiac surgery with CPB can prevent the deterioration of renal function. A multicenter prospective RCT in Japan reported that low-dose ANP infusion had no protective effect on kidney, but the finding was underpowered by its small sample size^[60]^. In general, the overall quality of evidence of the drugs was weak and conflicting.

Inotropic agents can be used to improve renal functions in low cardiac output states. Unlike other inotropes, levosimendan is a non-catecholamine calcium sensitizer which has stabilising effect on troponin C to enhance cardiac contractility without increasing myocardial oxygen consumption, leading to haemodynamic stability of myocardium^[61]^. It has been shown to have beneficial effect in decreasing low cardiac output syndrome and mortality after cardiac surgery^[62]^. An up-to-date meta-analysis of 12 RCTs conducted by Ng et al. reported that patients with preoperative low left ventricular ejection fraction (≤ 50%) who received levosimendan had significantly lesser risk of AKI than those treated with placebo^[63]^. Despite being reported as the most efficacious inodilator, levosimendan is, nonetheless, expensive and may not be available or ready to use in every centre.

Apart from the hematopoietic effect, studies have supported the idea that exogenous erythropoietin (EPO) exerts renoprotective effects against ischemia-reperfusion injury, suppressing oxidative stress and inflammation and attenuating apoptosis^[64]^. However, recent studies show conflicting findings. NMA conducted by Chen et al. showed that high-dose EPO (400500 IU/kg) did not have effect on AKI prevention after cardiac surgery but low-dose EPO (200-300 IU/kg) might be effective^[57]^. Another recent meta-analysis conducted by Penny-Dimri et al.^[65]^ found out a direct correlation between pre-anaesthetic EPO administration with reduced incidence of AKI postoperatively, but the authors claimed that the study yielded small sample size and demonstrated significant heterogeneity. Therefore, future larger studies are needed to ascertain the optimal dose and the timing of administration of EPO.

N-acetylcysteine (NAC) is a thiol compound which possesses antioxidant property that can scavenge ROS. Therefore, NAC can potentially ameliorate ischemia-reperfusion injury and inflammation, which are the main pathophysiology in CPB. NAC has also been demonstrated to decrease the risk of CIN in humans^[65]^. Although NAC is inexpensive, there is no solid evidence supporting the prophylactic administration of NAC in reducing incidence of AKI after cardiac surgery. One recent metaanalysis concluded that NAC was not effective in preventing CSA-AKI occurrence and improving SCr level or reducing length of hospital stay and mortality rate compared with placebo^[66]^.

KDIGO recommends intravenous saline or sodium bicarbonate for those who are at risk of CI-AKI^[20]^. However, a comprehensive systematic review which included 3563 patients found no clear evidence of benefits associated with the use of sodium bicarbonate^[67]^. While the pilot study of Hasse et al.^[68]^ found out that sodium bicarbonate infusion could significantly reduce the risk of AKI in patients undergoing cardiac surgery with CPB via urinary alkalization, recent meta-analyses failed to show the association of sodium bicarbonate infusion in lowering incidence of AKI, with one of the studies showing the possibility of increased mortality^[69]^.

Mannitol is an osmotic diuretic which is often added to CPB prime. Even though mannitol has been studied and used in the belief that it has renoprotective property in patients undergoing cardiac surgery, the evidence of benefit of mannitol in this setting remains controversial and contradictory^[68,70]^. In a prospective observational study, Bragadottir et al. reported that mannitol induces a renal vasodilation, which increases blood flow to the kidneys with balanced renal oxygenation and filtration fraction^[70]^. On the other hand, other studies found no differences between the mannitol group and the control cohort^[7]^.

Usually, volatile anaesthetics are used in surgery for general anaesthesia to induce and maintain analgesia, hypnosis, amnesia, and create mild relaxation of muscle. Besides, an animal study conducted by Jia et al. proved that volatile anaesthetics confer protection against renal ischemia-reperfusion injury^[71,72]^. Cai et al. performed a meta-analysis of 10 RCTs involving 1600 patients who underwent cardiac surgery^[73]^. Table 6 is a summary of their findings; the authors found out that administration of a volatile anaesthetic significantly reduced the incidence of postcardiovascular surgery AKI, as well as the incidence of prolonged stay in hospital/ICU, and slightly decreased mortality^[73]^.

**Table 6:** Main findings from Cai et al.^[74]^ study in relation to pharmacological protection of kidneys to prevent acute kidney injury (AKI) after cardiac surgery.

Pharmacological agents	Author/Year	Number of included trials	Total number of patients	Outcome	Pooled effect size (OR, 95% CI)	*P-*value	*I*^2^ (heterogeneity)
Dexmedetomidine	Liu et al., 2018	10	1575	AKI	0.65 (0.45, 0.92)	0.02	0%
Statin	Putzu et al., 2016	23	5102	AKI	1.26 (1.05, 1.52)	0.01	Not reported
Statin	Lewicki et al., 2015	7	662	AKI	RR 0.76 (0.46, 1.28)	Not reported	0%
Fenoldopam	Gillies et al., 2015	6	507	AKI	0.46 (0.27, 0.79)	0.004	0%
Fenoldopam	Zangrillo et al., 2012	6	440	AKI	0.41 (0.23, 0.74)	0.003	0%
Levosimendan	Ng et al., 2019	12	1867	AKI	0.61 (0.40, 0.92)	0.02	33%
Levosimendan	Tena et al., 2018	14	2243	RRT	RR 0.66 (0.47, 0.92)	0.015	0%
Erythropoietin	Penny-Dimri et al., 2016	6	473	AKI	0.69 (0.35, 1.36)	0.28	64%
N-acetylcysteine	Mei et al., 2017	10	1391	AKI	0.841 (0.691, 1.023)	0.116	39.4%
N-acetylcysteine	Ho et al., 2009	10	1193	AKI	1.04 (0.45, 2.37)	Not reported	3.3%
N-acetylcysteine	Nigwekar et al., 2009	12	1324	AKI	0.89 (0.68, 1.15)	0.36	0%
Sodium bicarbonate	Tie et al., 2014	5	1079	AKI	0.99 (0.78, 1.24)	0.911	56.1%
Sodium bicarbonate	Bailey et al., 2015	3	877	*	1.11 (0.77, 1.60)	0.45	Not reported
Sodium bicarbonate	Kim et al., 2015	5	1092	AKI	0.95 (0.74, 1.22)	0.71	59%
Sodium bicarbonate	Zoungas et al., 2009	23	3563	CIN	0.62 (0.45, 0.86)	Not reported	49.1%
Volatile anaesthetics	Cai et al., 2014	10	1600	AKI	0.65 (0.43, 0.97)	0.04	0%

* Postoperative increase in serum creatinine concentration of greater than 25% or 0.5 mg/dL within the first five postoperative days.

CI=confidence interval; CIN=contrast-induced nephropathy; OR=odds ratio; RR=risk ratio; RRT=renal replacement therapy

## CONCLUSION

CSA-AKI is a serious complication and can dramatically increase perioperative morbidity and mortality, even so, this condition can be prevented through different perioperative measures. Focused studies on identifying key predictive factors for such morbidity and monitoring the progress are crucial to improve outcomes in such high-risk cohort.

**Table:** 

Authors' roles & responsibilities	
AH	Substantial contributions to the conception or design of the work; or the acquisition, analysis, or interpretation of data for the work; drafting the work or revising it critically for important intellectual content; agreement to be accountable for all aspects of the work in ensuring that questions related to the accuracy or integrity of any part of the work are appropriately investigated and resolved; final approval of the version to be published
MJ	Substantial contributions to the conception or design of the work; or the acquisition, analysis, or interpretation of data for the work; drafting the work or revising it critically for important intellectual content; agreement to be accountable for all aspects of the work in ensuring that questions related to the accuracy or integrity of any part of the work are appropriately investigated
SG	Substantial contributions to the conception or design of the work; or the acquisition, analysis, or interpretation of data for the work; drafting the work or revising it critically for important intellectual content; agreement to be accountable for all aspects of the work in ensuring that questions related to the accuracy or integrity of any part of the work are appropriately investigated and resolved; final approval of the version to be published
WYT	Substantial contributions to the conception or design of the work; or the acquisition, analysis, or interpretation of data for the work; drafting the work or revising it critically for important intellectual content; agreement to be accountable for all aspects of the work in ensuring that questions related to the accuracy or integrity of any part of the work are appropriately investigated and resolved; final approval of the version to be published
FG	Substantial contributions to the conception or design of the work; or the acquisition, analysis, or interpretation of data for the work; drafting the work or revising it critically for important intellectual content; agreement to be accountable for all aspects of the work in ensuring that questions related to the accuracy or integrity of any part of the work are appropriately investigated and resolved; final approval of the version to be published
MS	Substantial contributions to the conception or design of the work; or the acquisition, analysis, or interpretation of data for the work; drafting the work or revising it critically for important intellectual content; agreement to be accountable for all aspects of the work in ensuring that questions related to the accuracy or integrity of any part of the work are appropriately investigated and resolved; final approval of the version to be published.

## References

[1] Mao H, Katz N, Ariyanon W, Blanca-Martos L, Adybelli Z, Giuliani A (2014). Cardiac surgery-associated acute kidney injury. Blood Purif.

[2] Bellomo R, Auriemma S, Fabbri A, D'Onofrio A, Katz N, McCullough PA (2008). The pathophysiology of cardiac surgery-associated acute kidney injury (CSA-AKI). Int J Artif Organs.

[3] Hobson CE, Yavas S, Segal MS, Schold JD, Tribble CG, Layon AJ (2009). Acute kidney injury is associated with increased long-term mortality after cardiothoracic surgery. Circulation.

[4] Yuan SM (2019). Acute kidney injury after pediatric cardiac surgery. Pediatr Neonatol.

[5] Lopes JA, Jorge S (2013). The RIFLE and AKIN classifications for acute kidney injury: a critical and comprehensive review. Clin Kidney J.

[6] Kellum JA, Lameire N, Aspelin P, Barsoum RS, Burdmann EA, Goldstein SL (2012). Kidney disease: improving global outcomes (KDIGO) acute kidney injury work group. KDIGO clinical practice guideline for acute kidney injury. Kid Int Suppl.

[7] O'Neal JB, Shaw AD, Billings FT 4th (2016). Acute kidney injury following cardiac surgery: current understanding and future directions. Critical Care.

[8] Rosner MH, Okusa MD (2006). Acute kidney injury associated with cardiac surgery. Clin J Am Soc Nephrol.

[9] Thomas ME, Blaine C, Dawnay A, Devonald MA, Ftouh S, Laing C (2015). The definition of acute kidney injury and its use in practice. Kidney Int.

[10] Stafford-Smith M, Li YJ, Mathew JP, Li YW, Ji Y, Phillips-Bute BG (2015). Genome-wide association study of acute kidney injury after coronary bypass graft surgery identifies susceptibility loci. Kidney Int.

[11] Lu JC, Coca SG, Patel UD, Cantley L, Parikh CR (2009). Translational Research Investigating Biomarkers and Endpoints for Acute Kidney Injury (TRIBE-AKI) Consortium. Searching for genes that matter in acute kidney injury: a systematic review. Clin J Am Soc Nephrol.

[12] Liu Y, Li H, Chen S, Chen J, Tan N, Zhou Y (2016). Excessively high hydration volume may not be associated with decreased risk of contrast-induced acute kidney injury after percutaneous coronary intervention in patients with renal insufficiency. J Am Heart Assoc.

[13] Meersch M, Schmidt C, Zarbock A. (2017). Perioperative acute kidney injury: an under-recognized problem. Anesth Analg.

[14] Obialo C (2017). Acute kidney injury following cardiopulmonary bypass surgery. Niger J Cardiovasc Thorac Surg.

[15] Cheungpasitporn W, Thongprayoon C, Kittanamongkolchai W, Srivali N, O'Corragain OA, Edmonds PJ (2015). Comparison of renal outcomes in off-pump versus on-pump coronary artery bypass grafting: a systematic review and meta-analysis of randomized controlled trials. Nephrology (Carlton).

[16] Lamy A, Devereaux PJ, Prabhakaran D, Taggart DP, Hu S, Paolasso E (2012). Off-pump or on-pump coronary-artery bypass grafting at 30 days. N Engl J Med.

[17] Bruins P, te Velthuis H, Yazdanbakhsh AP, Jansen PG, van Hardevelt FW, de Beaumont EM (1997). Activation of the complement system during and after cardiopulmonary bypass surgery: postsurgery activation involves c-reactive protein and is associated with postoperative arrhythmia. Circulation.

[18] Pleština S, Gamulin S (2001). Kidney ischaemia-reperfusion injury and polyribosome structure. Nephron.

[19] Khan UA, Coca SG, Hong K, Koyner JL, Garg AX, Passik CS (2014). Blood transfusions are associated with urinary biomarkers of kidney injury in cardiac surgery. J Thorac Cardiovasc Surg.

[20] Khwaja A (2012). KDIGO clinical practice guidelines for acute kidney injury. Nephron Clin Pract.

[21] Parikh CR, Thiessen-Philbrook H, Garg AX, Kadiyala D, Shlipak MG, Koyner JL (2013). Performance of kidney injury molecule-1 and liver fatty acid-binding protein and combined biomarkers of AKI after cardiac surgery. Clin J Am Soc Nephrol.

[22] Meersch M, Schmidt C, Van Aken H, Martens S, Rossaint J, Singbartl K (2014). Urinary TIMP-2 and IGFBP7 as early biomarkers of acute kidney injury and renal recovery following cardiac surgery. PLoS One.

[23] Haase-Fielitz A, Bellomo R, Devarajan P, Story D, Matalanis G (2009). Novel and conventional serum biomarkers predicting acute kidney injury in adult cardiac surgery--a prospective cohort study. Crit Care Med.

[24] Medalion B, Cohen H, Assali A, Vaknin Assa H, Farkash A, Snir E (2010). The effect of cardiac angiography timing, contrast media dose, and preoperative renal function on acute renal failure after coronary artery bypass grafting. J Thorac Cardiovasc Surg.

[25] Karkouti K, Wijeysundera DN, Yau TM, McCluskey SA, van Rensburg A, Beattie WS (2008). The influence of baseline hemoglobin concentration on tolerance of anemia in cardiac surgery. Transfusion.

[26] Walsh M, Garg AX, Devereaux PJ, Argalious M, Honar H, Sessler DI (2013). The association between perioperative hemoglobin and acute kidney injury in patients having noncardiac surgery. Anesth Analg.

[27] Reents W, Hilker M, Börgermann J, Albert M, Plötze K, Zacher M (2014). Acute kidney injury after on-pump or off-pump coronary artery bypass grafting in elderly patients. Ann Thorac Surg.

[28] van den Berghe G, Wouters P, Weekers F, Verwaest C, Bruyninckx F, Schetz M (2001). Intensive insulin therapy in critically ill patients. N Engl J Med.

[29] Song JW, Shim JK, Yoo KJ, Oh SY, Kwak YL (2013). Impact of intraoperative hyperglycaemia on renal dysfunction after off-pump coronary artery bypass. Interact Cardiovasc Thorac Surg.

[30] Thomson R, Meeran H, Valencia O, Al-Subaie N (2014). Goal-directed therapy after cardiac surgery and the incidence of acute kidney injury. J Crit Car.

[31] Magruder JT, Crawford TC, Harness HL, Grimm JC, Suarez-Pierre A, Wierschke C (2017). A pilot goal-directed perfusion initiative is associated with less acute kidney injury after cardiac surgery. J Thorac Cardiovasc Surg.

[32] Scott NB, Turfrey DJ, Ray DA, Nzewi O, Sutcliffe NP, Lal AB (2001). A prospective randomized study of the potential benefits of thoracic epidural anesthesia and analgesia in patients undergoing coronary artery bypass grafting. Anesth Analg.

[33] Svircevic V, Passier MM, Nierich AP, van Dijk D, Kalkman CJ, van der Heijden GJ (2013). Epidural analgesia for cardiac surgery. Cochrane Database Syst Rev.

[34] Landoni G, Isella F, Greco M, Zangrillo A, Royse CF (2015). Benefits and risks of epidural analgesia in cardiac surgery. Br J Anaesth.

[35] Xie J, Zhang X, Xu J, Zhang Z, Klingensmith NJ, Liu S (2018). Effect of remote ischemic preconditioning on outcomes in adult cardiac surgery: a systematic review and meta-analysis of randomized controlled studies. Anesth Analg.

[36] Zarbock A, Kellum JA, Van Aken H, Schmidt C, Küllmar M, Rosenberger P (2017). Long-term effects of remote ischemic preconditioning on kidney function in high-risk cardiac surgery patients: follow-up results from the renalRIP trial. Anesthesiology.

[37] Martin GS, Bassett P (2019). Crystalloids vs. colloids for fluid resuscitation in the intensive care unit: a systematic review and meta-analysis. J Crit Care.

[38] Young P, Bailey M, Beasley R, Henderson S, Mackle D, McArthur C (2015). Effect of a buffered crystalloid solution vs. saline on acute kidney injury among patients in the intensive care unit: the SPLIT randomized clinical trial.. Jama.

[39] Krajewski ML, Raghunathan K, Paluszkiewicz SM, Schermer CR, Shaw AD (2015). Meta-analysis of high- versus low-chloride content in perioperative and critical care fluid resuscitation. Br J Surg.

[40] Myburgh JA, Finfer S, Bellomo R, Billot L, Cass A, Gattas D (2012). Hydroxyethyl starch or saline for fluid resuscitation in intensive care. N Engl J Med.

[41] Soh S, Song JW, Shim JK, Kim JH, Kwak YL (2016). Sodium bicarbonate does not prevent postoperative acute kidney injury after off-pump coronary revascularization: a double-blinded randomized controlled trial. Br J Anaesth.

[42] Haase-Fielitz A, Haase M, Bellomo R, Calzavacca P, Spura A, Baraki H (2017). Perioperative hemodynamic instability and fluid overload are associated with increasing acute kidney injury severity and worse outcome after cardiac surgery. Blood Purif.

[43] Bhaskaran K, Arumugam G, Vinay Kumar PV (2018). A prospective, randomized, comparison study on effect of perioperative use of chloride liberal intravenous fluids versus chloride restricted intravenous fluids on postoperative acute kidney injury in patients undergoing off-pump coronary artery bypass grafting surgeries. Ann Card Anaesth.

[44] Shen Y, Zhang W, Cheng X, Ying M (2018). Association between postoperative fluid balance and acute kidney injury in patients after cardiac surgery: a retrospective cohort study. J Crit Care.

[45] Benedetto U, Luciani R, Goracci M, Capuano F, Refice S, Angeloni E (2009). Miniaturized cardiopulmonary bypass and acute kidney injury in coronary artery bypass graft surgery. Ann Thorac Surg.

[46] Chew ST, Ng RR, Liu W, Goh SG, Caleb MG, Ti LK (2016). Miniaturized versus conventional cardiopulmonary bypass and acute kidney injury after cardiac surgery. Perfusion.

[47] Karim H, Yunus M, Saikia M, Kalita J, Mandal M (2017). Incidence and progression of cardiac surgery-associated acute kidney injury and its relationship with bypass and cross clamp time. Ann Card Anaesth.

[48] Kumar AB, Suneja M, Bayman EO, Weide GD, Tarasi M (2012). Association between postoperative acute kidney injury and duration of cardiopulmonary bypass: a meta-analysis. J Cardiothorac Vasc Anesth.

[49] Träger K, Skrabal C, Fischer G, Datzmann T, Schroeder J, Fritzler D (2017). Hemoadsorption treatment of patients with acute infective endocarditis during surgery with cardiopulmonary bypass - a case series. Int J Artif Organs.

[50] Stewart RM, Park PK, Hunt JP, McIntyre RC Jr, McCarthy J, Zarzabal LA (2009). Less is more: improved outcomes in surgical patients with conservative fluid administration and central venous catheter monitoring. J Am Coll Surg.

[51] Sun S, Ma F, Li Q, Bai M, Li Y, Yu Y (2017). Risk model for deaths and renal replacement therapy dependence in patients with acute kidney injury after cardiac surgery. Interact Cardiovasc Thorac Surg.

[52] Palevsky PM (2013). Renal replacement therapy in acute kidney injury. Adv Chronic Kidney Dis.

[53] Liu Y, Sheng B, Wang S, Lu F, Zhen J, Chen W (2018). Dexmedetomidine prevents acute kidney injury after adult cardiac surgery: a meta-analysis of randomized controlled trials. BMC Anesthesiol.

[54] Balkanay OO, Goksedef D, Omeroglu SN, Ipek G (2015). The dose-related effects of dexmedetomidine on renal functions and serum neutrophil gelatinase-associated lipocalin values after coronary artery bypass grafting: a randomized, triple-blind, placebo-controlled study. Interact Cardiovasc Thorac Surg.

[55] 4th Billings FT, Pretorius M, Siew ED, Yu C, Brown NJ (2010). Early postoperative statin therapy is associated with a lower incidence of acute kidney injury after cardiac surgery. J Cardiothorac Vasc Anesth.

[56] Lewicki M, Ng I, Schneider AG (2015). HMG CoA reductase inhibitors (statins) for preventing acute kidney injury after surgical procedures requiring cardiac bypass. Cochrane Database Syst Rev.

[57] Chen X, Huang T, Cao X, Xu G (2018). Comparative efficacy of drugs for preventing acute kidney injury after cardiac surgery: a network meta-analysis. Am J Cardiovasc Drugs.

[58] Ranucci M, De Benedetti D, Bianchini C, Castelvecchio S, Ballotta A, Frigiola A (2010). Effects of fenoldopam infusion in complex cardiac surgical operations: a prospective, randomized, double-blind, placebo-controlled study. Minerva Anestesiol.

[59] Gillies MA, Kakar V, Parker RJ, Honoré PM, Ostermann M (2015). Fenoldopam to prevent acute kidney injury after major surgery-a systematic review and meta-analysis. Critical Care.

[60] Sezai A, Hata M, Niino T, Yoshitake I, Unosawa S, Wakui S (2009). Influence of continuous infusion of low-dose human atrial natriuretic peptide on renal function during cardiac surgery: a randomized controlled study. J Am Coll Cardiol.

[61] Mitaka C, Si MK, Tulafu M, Yu Q, Uchida T, Abe S (2014). Effects of atrial natriuretic peptide on inter-organ crosstalk among the kidney, lung, and heart in a rat model of renal ischemia-reperfusion injury. Intensive Care Med Exp.

[62] Ng KT, Chan XL, Tan W, Wang CY (2019). Levosimendan use in patients with preoperative low ejection fraction undergoing cardiac surgery: a systematic review with meta-analysis and trial sequential analysis. J Clin Anesth.

[63] Tena MÁ, Urso S, González JM, Santana L, Sadaba R, Juarez P (2018). Levosimendan versus placebo in cardiac surgery: a systematic review and meta-analysis. Interact Cardiovasc Thorac Surg.

[64] Sølling C, Christensen AT, Krag S, Frøkiaer J, Wogensen L, Krog J (2011). Erythropoietin administration is associated with short-term improvement in glomerular filtration rate after ischemia-reperfusion injury. Acta Anaesthesiol Scand.

[65] Penny-Dimri JC, Cochrane AD, Perry LA, Smith JA (2016). Characterising the role of perioperative erythropoietin for preventing acute kidney injury after cardiac surgery: systematic review and meta-analysis. Heart Lung Circ.

[66] Kelly AM, Dwamena B, Cronin P, Bernstein SJ, Carlos RC (2008). Meta-analysis: effectiveness of drugs for preventing contrast-induced nephropathy. Ann Intern Med.

[67] Mei M, Zhao HW, Pan QG, Pu YM, Tang MZ, Shen BB (2018). Efficacy of N-Acetylcysteine in preventing acute kidney injury after cardiac surgery: a meta-analysis study. J Invest Surg.

[68] Haase M, Haase-Fielitz A, Bellomo R, Devarajan P, Story D, Matalanis G (2009). Sodium bicarbonate to prevent increases in serum creatinine after cardiac surgery: a pilot double-blind, randomized controlled trial. Crit Care Med.

[69] Zoungas S, Ninomiya T, Huxley R, Cass A, Jardine M, Gallagher M (2009). Systematic review: sodium bicarbonate treatment regimens for the prevention of contrast-induced nephropathy. Ann Intern Med.

[70] Kim JH, Kim HJ, Kim JY, Hs Ahn, Ahn IM, Choe WJ (2015). Meta-analysis of sodium bicarbonate therapy for prevention of cardiac surgery-associated acute kidney injury. J Cardiothorac Vasc Anesth.

[71] Bragadottir G, Redfors B, Ricksten SE (2012). Mannitol increases renal blood flow and maintains filtration fraction and oxygenation in postoperative acute kidney injury: a prospective interventional study. Crit Care.

[72] Yallop KG, Sheppard SV, Smith DC (2008). The effect of mannitol on renal function following cardio-pulmonary bypass in patients with normal pre-operative creatinine. Anaesthesia.

[73] Jia P, Teng J, Zou J, Fang Y, Zhang X, Bosnjak ZJ (2013). miR-21 contributes to xenon-conferred amelioration of renal ischemia-reperfusion injury in mice. Anesthesiology.

[74] Cai J, Xu R, Yu X, Fang Y, Ding X (2014). Volatile anesthetics in preventing acute kidney injury after cardiac surgery: a systematic review and meta-analysis. J Thorac Cardiovasc Surg.

